# Resilience as a Factor Influencing Psychological Distress Experience in Patients with Neuro-Oncological Disease

**DOI:** 10.3390/curroncol29120776

**Published:** 2022-12-15

**Authors:** Jan Ilgen, Mirjam Renovanz, Andreas Stengel, Stephan Zipfel, Norbert Schäffeler

**Affiliations:** 1 Department of Psychosomatic Medicine, and Psychotherapy, Tübingen University Hospital, 72076 Tübingen, Germany; 2 Department of Neurology and Interdisciplinary Neuro-Oncology, Hertie Institute for Clinical Brain Research, Eberhard Karls University of Tübingen, 72076 Tübingen, Germany; 3 Center for Neuro-Oncology, Comprehensive Cancer Center Tübingen-Stuttgart, Tübingen University Hospital, Eberhard Karls University of Tübingen, 72076 Tübingen, Germany; 4 Department of Neurosurgery, Tübingen University Hospital, Eberhard Karls University Tübingen, 72076 Tübingen, Germany; 5 Psycho-Oncology Section, Comprehensive Cancer Center Tübingen-Stuttgart, Tübingen University Hospital, 72076 Tübingen, Germany; 6 Charité Center for Internal Medicine and Dermatology, Department of Psychosomatic Medicine, Charité-Universitätsmedizin Berlin, Corporate Member of Freie Universität Berlin, Humboldt-Universität Berlin and Berlin Institute of Health, 10117 Berlin, Germany

**Keywords:** distress, psycho-oncology, neuro-oncology, resilience, screening

## Abstract

Cancer causes psychological distress. Approximately one-third of all patients with cancer suffer from distress requiring psycho-oncological treatment. Examining factors contributing to their distress can inform approaches to counteracting them. Among such factors, resilience is considered to be a psychological adaptive capacity resulting from complex genetic, epigenetic, psychological, and environmental influences. For that reason, we investigated resilience as a factor of psychological distress experience among patients with neuro-oncological disease. To assess distress among patients with neuro-oncological diseases, we performed electronic psycho-oncological screening in the Department of Neurosurgery at Tübingen University Hospital (*n* = 100) following tumor surgery (T_0_) using the Resilience Scale 13, the Hornheider Screening Instrument, the Patient Health Questionnaire-2, the Generalized Anxiety Disorder Scale-2, and the Distress Thermometer, all administered on tablets. Follow-up was done 6 months after (T_1_). The distress of patients with neuro-oncological disease decreased significantly after 6 months (*p* < 0.01). Most patients (87%) showed moderate to high resilience. Although significant correlations with distress are measurable at the T_0_ time point (ρ = −0.318 **, *p* < 0.01), no significant correlations were observed at T_1_. Thus, resilience seems to significantly impact distress in the acute phase of the neuro-oncological disease. For clinical practice, our findings suggest that resilience-focused screening can provide useful information about patients at risk of experiencing distress.

## 1. Introduction

As a life-threatening disease, cancer typically prompts major changes in lifestyle, which can can cause distress. In recent decades, research has indeed shown the traumatic potential of cancer as a source of distress [1]. In context of psychology research, the term distress goes back to Hans Selye, pioneer in research on stress who coined the terms eustress and distress, meaning positively and negatively experienced stress, respectively [2]. Nevertheless, there is no scientifically uniform definition of distress. In this paper, distress refers to the experience of emotional strain. Various studies have shown a correlation between distress and cancer [3,4,5,6]. Because approximately one-third of all patients with cancer suffer from distress that requires psycho-oncological treatment [7,8,9,10], it is important to investigate factors of their distress in order to be able to offer them targeted help.

One such factor is resilience, a concept that has become increasingly important in psychotherapy and psychology in the past decade. The term resilience originates from the work of psychologist Jack Block, whose term ego-resilience describes being controlled as much as necessary but as little as possible, depending on the situation. Today, resilience is studied within and between disciplines and is therefore described in highly different contexts. Among other things, it is debated whether resilience is a state (i.e., temporary) or a trait (i.e., enduring) [11,12]. On the one hand, resilience can describe a form of adaptability that deals with maintaining or regaining normal functioning; on the other, authors have equated it with the ability to continue to develop despite adversity [11,13]. Along those lines, resilience is often characterized as a personality trait, especially in person-centered approaches [11]. Past studies have revealed resilience’s effects on distress and depressive symptoms in other patient populations [3,4,5,6,14].

Because neuro-oncological diseases often affect the CNS, they can sometimes involve not only the brain but also the spinal cord. Brain tumors are rare and can be divided into tumors of primary and secondary genesis [15]. The most common brain tumors of primary genesis include gliomas, meningiomas, and pituitary adenomas, all of which can be classified into the World Health Organization’s grades I–IV and thus as benign and malignant [16]. Surgery, chemotherapy, and radiotherapy are often mainstays of treatment for such tumors [17]. At the same time, because brain metastases occur in approximately 20% of all patients with cancer [18], other tumors of primary genesis may also have a neuro-oncological component. Cerebral metastases thus also belong to the neuro-oncological spectrum of disease. In any case, neuro-oncological tumors are sometimes associated with a poor prognosis and a moderate to high risk of neurological disability, both of which results in an increased risk of distress, depression, and anxiety [19].

Against that background, we examined the impact of resilience on distress among patients with neuro-oncological disease—that is, with any tumors requiring neurosurgical therapy. We particularly investigated whether significant relationships exist between resilience, on the one hand, and, on the other, distress, anxiety, depression, and the need for psychosocial support. We examined those relationships in inpatient (T_0_) and outpatient settings 6 months after surgery (T_1_). Our hypothesis was that higher levels of resilience are associated with lower levels of distress, depressive symptoms, anxiety symptoms, and need for psychosocial support at T_0_ and T_1_.

## 2. Materials and Methods

### 2.1. Sample

From February 2018 to February 2019, we examined patients who were undergoing surgical tumor therapy in the Department of Neurosurgery at Tübingen University Hospital. We asked patients at admission as inpatients (T_0_) to complete routine psycho-oncological screening via a questionnaire encompassing five instruments. The inclusion criterion was needing surgical treatment at inpatient admission for a tumor of neuro-oncological origin (i.e., suspicion of a malignant tumor, recurring tumor, or second tumor). By contrast, the exclusion criteria were cognitive deficits (e.g., somnolence and pronounced aphasia) that make adequate oral conversation impossible. Although we tried to perform the screening with 140 patients, only 100 were ultimately included, for a participation rate of 71%. Six months later (T_1_), the same questionnaire was readministered over the phone, and 59% of the patients participated.

Overall, patients were 52 years old on average (*SD* = 14), and the sex distribution in the sample was largely even, with 53% of participants being female. The three most common tumor entities were meningiomas, gliomas, and metastases. Table 1 presents the participants’ demographic data, whereas Table 2 offers an overview of their neuro-oncological diseases. Furthermore Table 3 shows the participants’ disease status. The study was approved by the local ethics committee (No. 602/2014BO2).

### 2.2. Assessment

We administered a psycho-oncological distress screening via a questionnaire with five instruments between 1 and 3 days after neurosurgery and again 6 months later over the phone. The instruments were used to measure distress, need for psychosocial support, and symptoms of anxiety and depression, as described in the following subsections.

*The Resilience Scale 13 (RS-13)*, an instrument with 13 items for measuring resilience, is the short form of the RS-25 [20], with good psychometric properties [21]. The 13 items address personality traits (e.g., “Keeping interested in things is important to me” and “I am determined”) and are rated on a 7 point Likert scale ranging from 1 (*disagree*) to 7 (*agree*), for a total score subsequently used to calculate the level of resilience. Scores of at least 73 indicate a high level of resilience, whereas ones not exceeding 66 indicate a low level, such that scores ranging from 67 to 72 indicate a moderate level of resilience [21,22]. Cronbach’s alpha for the RS-13 in our sample was 0.823.

*The Distress Thermometer (DT)*, used to determine current subjective distress, has an 11 point visual analog scale ranging from 0 (*no distress*) to 10 (*extreme distress*), with 5, considered to indicate medium distress, used as a threshold value indicating a psycho-oncological need for counseling [23,24,25]. Studies have shown the DT is acceptable to very good validity [26].

*The Hornheider Screening Instrument (HSI),* was used to measure patients’ need for psycho-oncological support. Suitable for use during the initial contact between physicians and patients and characterized by good reliability and validity, the HSI assesses the physical and mental condition of patients with seven items using the answer categories *yes* and *no*. The items address the patients’ perceived burden of their disease on relatives, their contact persons in the case of worries and/or fears, and other perceived burdens independent of their disease [27].

*The Patient Health Questionnaire-2 (PHQ-2),* a short form of the PHQ-9 used to screen patients for depressive symptoms, is characterized by a high specificity for and high sensitivity to depression. Two items are used to inquire about patients’ loss of interest and joy, as well as dejection, which are regarded as leading symptoms of depression [28,29].

*The Generalized Anxiety Discorder Scale-2 (GAD-2),* the short form of GAD-7, consists of two items about anxiety and the feeling of being unable to stop or control worrying, with response options for frequency referring to the past 2 weeks (i.e., “Not at all,” “Several days,” “More than half of the days,” and “Nearly every day”) [30]. The GAD-2 is considered to have good psychometric properties in terms of specificity and sensitivity [31], and its simplicity makes it ideally suited for a briefly screening for anxiety.

### 2.3. Statistical Analyses

All statistical analyses were performed in SPSS version 25.0. Correlations were determined using Spearman’s rho (ρ), the significance in changes from T_0_ to T_1_ was calculated with the Wilcoxon signed-rank test, and between-group differences were calculated using the Kruskal–Wallis test, with significance set at *p* < 0.05.

## 3. Results

The participants’ descriptive psychometric data, shown in Table 4, indicate expressions of distress, a need for psychosocial support, depression, and anxiety. Table 4 also presents the degree of change in the 6 month period from T_0_ to T_1_.

### 3.1. Resilience

**Figure 1:**
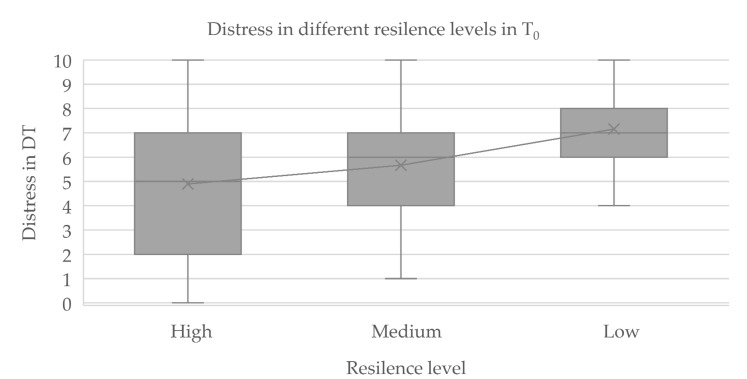
Distress in different resilience levels.

Per results on the RS-13, 87% of patients had moderate to high resilience (i.e., 66% with high resilience and 21% with moderate resilience), whereas 13% had low resilience. Table 5 shows the mean scores for distress, need for psychosocial support, depressive symptoms, and anxiety symptoms by level of resilience. Figure 1 illustrates the differences between the distress levels at T_0_ in the resilience-based groups in a bar chart. In addition, Table 6 presents correlations between resilience and distress, need for psychosocial support, depression, and anxiety as a crosstab.

Figure 1 shows three boxplots with the scores for distress in the different resilience-based groups. The upper whisker shows the maximum value and the lower whisker the minimum value, while the lower part of the box shows the first quartile and the upper part the third quartile. Mean values increased within the three resilience-based groups from 4.89 to 5.67 and to 7.15, respectively.

A significant negative correlation emerged between scores for resilience and distress on Spearman’s rho test at ρ = −0.318 ** (*p* < 0.01, Table 6) at T_0_. Moreover, the Kruskal–Wallis test revealed significant differences within the groups (*p* = 0.018). In the linear regression, *r*^2^ = 0.11 *** (*p* < 0.001), a significant difference appeared only between the groups with high and low resilience (*p* = 0.016, Figure 1), with a medium effect size of 0.33. The correlation between resilience and distress was no longer significant at T_1_, however.

### 3.2. Need for Psychosocial Support

Although a significant negative correlation emerged between scores for resilience and the need for psychosocial support (i.e., HSI scores) on Spearman’s rho test at ρ = −0.235 * (*p <* 0.05, Table 6) at T_0_, the correlation was no longer significant 6 months later.

### 3.3. Depression

No significant correlations emerged between resilience and depressive symptoms (i.e., PHQ-2 scores) at either T_0_ or T_1_.

### 3.4. Anxiety

Although a significant negative correlation emerged between resilience and anxiety symptoms (i.e., GAD-2 scores) on Spearman’s rho test at ρ = −0.323 ** (*p < 0*.01, Table 6) at T_0_, the correlation was no longer significant 6 months later.

## 4. Discussion

Our primary objective was to investigate distress and its factors among patients with neuro-oncological disease. Among our results, distress among the patients dropped in the 6 months following surgical tumor therapy, a rather brief period that few studies have measured distress within. Reasons for the significant decrease could be that the hospital atmosphere and postoperative pain negatively affected distress and/or that such patients’ ultimate diagnosis remains unclear until the final histological results are obtained. Along those lines, many patients in our study were suspected of having meningiomas, which tend to be benign [32]. As a result, a large proportion of patients did not end up with cancer and are in remission after surgery.

The expression of anxiety symptoms also decreased significantly in the 6 month interval, a result also found in other studies examining anxiety symptoms across similar periods of time [33]. This was not shown for depression symptoms. Depression is more common in cancer patients than in the normal population. The trend was not observed for depressive symptoms, however. Because depression is more common among patients with cancer than in the general population, patients with neuro-oncological diseases, as well as patients with other types of cancer [34], are at risk of suffering from depressive symptoms over relatively long periods, meaning that depression is a worse long-term side effect for them than anxiety [35]. It can also be concluded that anxiety symptoms decrease in intensity faster than depressive symptoms. Some authors have characterized anxiety symptoms as being relatively prominent at the time of diagnosis but depressive symptoms as being more likely after treatment [36]. Other studies have shown that elevated levels of anxiety and depression can be measured over longer periods during patients’ treatment [37]. Last, the need for psychosocial support did not decrease from T_0_ to T_1_, which suggests that such support should be provided to patients for more than 6 months after surgery.

A growing number of publications have linked resilience in patients with cancer to enhanced adaptation to symptoms of cancer, including distress and depression, and to improved quality of life [1,3]. Supported by our results, resilience thus seems to be an influential factor affecting experiences with distress among patients with neuro-oncological diseases, as was particularly evident among patients in our study at T_0_. Our results show that a high level of resilience can exert a protective influence on distress in hospital. This is an influence that decreases with time and can no longer be detected 6 months after surgery. That finding contributes to the rather limited knowledge about resilience among patients with neuro-oncological diseases. Beyond that, few studies have measured distress at multiple points in time, and ones that have done so have often examined intervals longer than 6 months. Even so, a significant relationship between resilience and distress has been shown in other studies and could also be observed in the population of patients that we studied [3,4,38,39,40].

Patients with low levels of resilience generally showed higher distress parameters on average. Thus, in clinical practice and psycho-oncological screening, measuring resilience can allow for identifying patients at risk of experiencing distress. Psychosocial interventions to strengthen resilience could thus be implemented to reduce distress [41].

Because we examined only patients with neuro-oncological tumors, our results are limited in their generalizability. Even then, their various tumor entities differed greatly in terms of prognosis and physical limitations, differences that we did not examine as variables potentially affecting experiences with distress. In future studies on the course of distress during patients’ treatment, tumor entities should be measured as a possible primary contributing factor to distress. Last, the group with low resilience was smaller than the other groups in our study. In a future study, a larger sample involving more patients with low levels of resilience should therefore be evaluated.

## 5. Conclusions

By examining resilience’s impact on distress, we have gained new insights into how distress is experienced in a patient population that has rarely been studied before—that is, patients with neuro-oncological disease. In particular, it appears that resilience exerts a significant impact in the acute phase of such diseases. For that reason, we recommend administering a resilience-oriented measure in the initial phase of hospitalization, which can provide useful information about distress as well as information to identify patients at risk of experiencing distress.

## Figures and Tables

**Table 1:** Participants‘ demographic data.

Demographic Data	
Total: *n* = 100	
Age	52 years (*SD* = 14)
Sex	
Female	*n* = 53 (53%)
Male	*n* = 47 (47%)
Relationship status	
Without partner	*n* = 10 (10%)
With Partner, but not married	*n* = 16 (16%)
Married	*n* = 68 (68%)
Separated	*n* = 3 (3%)
Divorced	*n* = 3 (3%)
Living situation	
Alone	*n* = 12 (12%)
With partner	*n* = 41 (41%)
Alone with children	*n* = 5 (5%)
With partner and children	*n* = 33 (33%)
With parents	*n* = 7 (7%)
In institution	*n* = 2 (2 %)
Children?	
No	*n* = 26 (26%)
Yes	*n* = 74 (74%)
Highest level of education	
Secondary (high) school	*n* = 69 (69%)
University	*n* = 22 (22%)
Other	*n* = 9 (9%)
Currently or past use of psychotropic medication?	
No	*n* = 87 (87%)
Yes	*n* = 13 (13%)
Current or former Psychotherapeutic or psychiatric treatment?	
No	*n* = 77 (77%)
Yes	*n* = 23 (23%)

**Table 2:** Tumor entities among participants.

Tumor Entity	*n* (%)
Meningeoma	35 (35)
Glioma	30 (30)
Metastasis	19 (19)
Unclear tumor	14 (14)
Other	2 (2)

**Table 3:** Participants’ disease status.

Status of Disease	*n* (%)
First episode Relapse, metastasis, secondary tumor, currently not assessable	75 (75%) 25 (25%)

**Table 4:** Descriptive statistics regarding distress, psychosocial support, anxiety, and depression at T_0_ and T_1_.

Instrument	Mean at T_0_ (*n* = 100)	Mean at T_1_ (*n* = 59)	Change (Wilcoxon Signed-Rank Test)
Distress Thermometer	5.35 (*SD* = 2.67)	3.90 (*SD* = 3.06)	*p* < 0.01
Hornheider Screening Instrument	0.014 (*SD* = 1.26)	−0.173 (*SD* = 1.58)	*p* = 0.312
Patient Health Questionnaire-2	1.46 (*SD* = 1.59)	1.00 (*SD* = 1.60)	*p* = 0.078
Generalized Anxiety Disorder Scale-2	1.67 (*SD* = 1.51)	0.78 (*SD* = 1.29)	*p* < 0.001

**Table 5:** Mean scores for distress, need for psychosocial support, depression, and anxiety by level of resilience.

Instrument	Low Resilience	Moderate Resilience	High Resilience
DT (T_0_)	7.15 (*SD* = 1.82)	5.67 (*SD* = 2.18)	4.89 (*SD* = 2.81)
DT (T_1_)	5.60 (*SD* = 1.95)	3.73 (*SD* = 2.80)	3.74 (*SD* = 3.22)
HSI (T_0_)	0.48 (*SD* = 1.37)	0.20 (*SD* = 1.37)	−0.14 (*SD* = 1.20)
HSI (T_1_)	0.95 (*SD* = 1.82)	−0.20 (*SD* = 1.49)	−0.30 (*SD* = 1.56)
PHQ-2 (T_0_)	2.08 (*SD* = 1.80)	1.43 (*SD* = 1.60)	1.35 (*SD* = 1.53)
PHQ-2 (T_1_)	0.56 (*SD* = 0.54)	0.36 (*SD* = 0.81)	1.20 (*SD* = 1.78)
GAD-2 (T_0_)	2.69 (*SD* = 1.49)	1.95 (*SD* = 1.43)	1.38 (*SD* = 1.46)
GAD-2 (T_1_)	1.80 (*SD* = 1.48)	0.45 (*SD* = 0.93)	0.74 (*SD* = 1.31)

Note: DT = Distress Thermometer; HSI = Hornheider Screening Instrument; PHQ-2 = Patient Health Questionnaire-2; GAD-2 = Generalized Anxiety Disorder Scale-2.

**Table 6:** Correlations between resilience and distress, need for psychosocial support, depression, and anxiety.

	DT		HSI		PHQ-2		GAD-2	
	T_0_	T_1_	T_0_	T_1_	T_0_	T_1_	T_0_	T_1_
**RS-13**	−0.318 **	/	−0.235 *	/	/	/	−0.323 **	/

Note: RS-13 = Resilience Scale 13; DT = Distress Thermometer; HSI = Hornheider Screening Instrument; PHQ-2 = Patient Health Questionnaire-2; GAD-2 = Generalized Anxiety Disorder Scale-2; * = *p* < 0.05; ** = *p* < 0.01.

## Data Availability

The data that support the findings of this study are available on request from the corresponding author. The data are not publicly available due to privacy or ethical restrictions.
